# Malaria vector dynamics and utilization of insecticide-treated nets in low-transmission setting in Southwest Ethiopia: implications for residual transmission

**DOI:** 10.1186/s12879-021-06592-9

**Published:** 2021-08-28

**Authors:** Endalew Zemene, Denekew Bitew Belay, Abebaw Tiruneh, Ming-Chieh Lee, Delenasaw Yewhalaw, Guiyun Yan

**Affiliations:** 1 School of Medical Laboratory Sciences, Institute of Health, Jimma University, Jimma, Ethiopia; 2 Department of Statistics, College of Science, Bahir Dar University, Bahir Dar, Ethiopia; 3 Program in Public Health, College of Health Sciences, University of California at Irvine, Irvine, CA 92697 USA; 4 Tropical and Infectious Diseases Research Center, Jimma University, Jimma, Ethiopia

**Keywords:** Anopheline vector dynamics, Human behaviour, ITNs, Residual malaria transmission, Ethiopia

## Abstract

**Background:**

Understanding the behaviour of local malaria vectors is essential as effectiveness of the commonly used vector-targeted malaria control tools heavily relies on behaviour of the major malaria vectors. This study was conducted to determine species composition, biting behaviour, host preference and infectivity of anopheline mosquitoes, and assess utilization of insecticide-treated nets (ITNs) in a low transmission setting in Southwest Ethiopia.

**Methods:**

Adult anopheline mosquitoes were collected using human landing catches (HLCs), Centers for Disease Control and Prevention (CDC) light traps (LTs) and Pyrethrum Spray Catches (PSCs) from June 2016 to May 2018 in Kishe, Jimma Zone, Southwest Ethiopia. The anopheline mosquitoes were morphologically identified. Moreover, sub-sample of *An. gambiae* s.l. was identified to species using polymerase chain reaction (PCR). Circum-sporozoite proteins (CSPs) and blood meal sources of the anopheline mosquitoes were tested using enzyme-linked immunosorbent assay (ELISA). In addition, a cross-sectional survey was conducted to assess ITN utilization by the inhabitants.

**Results:**

A total of 3659 anopheline mosquitoes comprising *An. coustani* complex (84.4%), *An. gambiae* s.l. (11.3%), and *An. pharoensis* and *An. squamosus* comprising less than 5% were collected. The anopheline mosquitoes showed marked outdoor (67%) and early evening (63%) biting behaviour. *An. coustani* complex and *An. gambiae* s.l. were predominantly zoophilic and anthropophilic, respectively. None of the sampled anopheline were CSP-positive. Most of the households (97.8%) owned at least one ITN, with modest usage by the inhabitants (73.4%). ITN usage was significantly higher among under-five children (AOR = 7.9, 95% CI: 4.41–14.03), household heads and spouses (AOR = 4.8, 95% CI: 3.0–7.59), those with sufficient access to ITNs (AOR = 1.8, 95% CI: 1.39–2.35), and who were not utilizing alternative mosquito repellents (AOR = 2.2, 95% CI: 1.58–2.99).

**Conclusion:**

The anopheline mosquito species exhibited predominantly outdoor and early evening biting activity. Household ITN coverage was high with slight gap in usage. Vector control interventions should target outdoor and early biting vectors to further suppress the local mosquito population. Moreover, sensitization of the community on consistent use of ITNs is required.

**Supplementary Information:**

The online version contains supplementary material available at 10.1186/s12879-021-06592-9.

## Background

After failure of the global malaria eradication program in the second half of the last century, a renewed effort of malaria eradication has been initiated in recent years. As a result, several countries have recently been declared malaria-free [1]. Globally, remarkable success in malaria control has been achieved during the Millennium Development Goals, mainly as a result of malaria interventions [2]. The successes achieved in the control of malaria were mainly attributable to availability of potent antimalarial drugs (particularly artemisinin-based combination therapy) and deployment indoor residual spraying (IRS) and long-lasting insecticidal nets (LLINs). These interventions have also substantially contributed to the control of malaria in Ethiopia. Nevertheless, malaria still remains disease of significant public health and socio-economic concern in underdeveloped part of the world, with few countries carrying majority of the global burden of malaria [3].

In Ethiopia, malaria control mainly relies on passive case detection and treatment of confirmed cases, and use of key vector control interventions. While IRS is conducted annually, as most malaria transmission areas experience seasonal transmission, LLINs are mass-distributed to malaria-at-risk communities every three years. The vector control interventions, introduction of the artemisinin-based combination therapy and increased access to malaria diagnostics have remarkably contributed to the decline in morbidity and malaria-related mortality achieved over the last decade in Ethiopia [4, 5]. Despite the role of these vector control interventions in the control of malaria, evidence of behaviour of the malaria vectors particularly in perceived low-transmission settings in Ethiopia is scarce.

Effectiveness of the vector control tools heavily relies on evidence of behaviour of local malaria vectors. Long-term application of the vector-directed control interventions may also alter biting behaviour of local mosquito vectors [6, 7], which has implications on malaria transmission. Despite the historical account of malaria in Kishe area [8], and long-term application of vector control interventions, entomological indices of anopheline mosquitoes in the area are not known.

Several indices can be utilised to measure malaria transmission intensity in a particular area. However, entomological inoculation rate (EIR) is the most direct measure for malaria transmission intensity. Entomological inoculation rate may also be used to evaluate effectiveness of malaria vector control interventions, as successful vector control interventions reduce the EIR [9]. The same species of *Anopheles* mosquito may display spatial heterogeneity in its preferred biting time, host preference, biting location and resting venue [10, 11], and some of these phenotypic plasticity could be induced by vector control interventions [6, 12]. These behavioural aspects of the major malaria vectors largely dictate the type of vector control interventions to be deployed. Kishe area, despite one of the malarious areas in Jimma Zone, presence of small-scale irrigation activities and several years of deployment of malaria vector control interventions, the status of malaria transmission in the area is not known. Annual IRS has been used for malaria control in the area for the last ten years. Moreover, LLINs have been distributed to the households every three years through the health extension program. The LLINs were provided to the households proportional to their family size, approximately one LLIN to every two family members. In this study, dynamics of anopheline mosquito species was monitored monthly to determine the species composition, host preference, biting cycles, preferred biting location and infectivity rate. Moreover, some of the human behavioural factors predisposing to mosquito bite, and utilization of ITNs were assessed.

## Methods

### Study setting

**Fig. 1:**
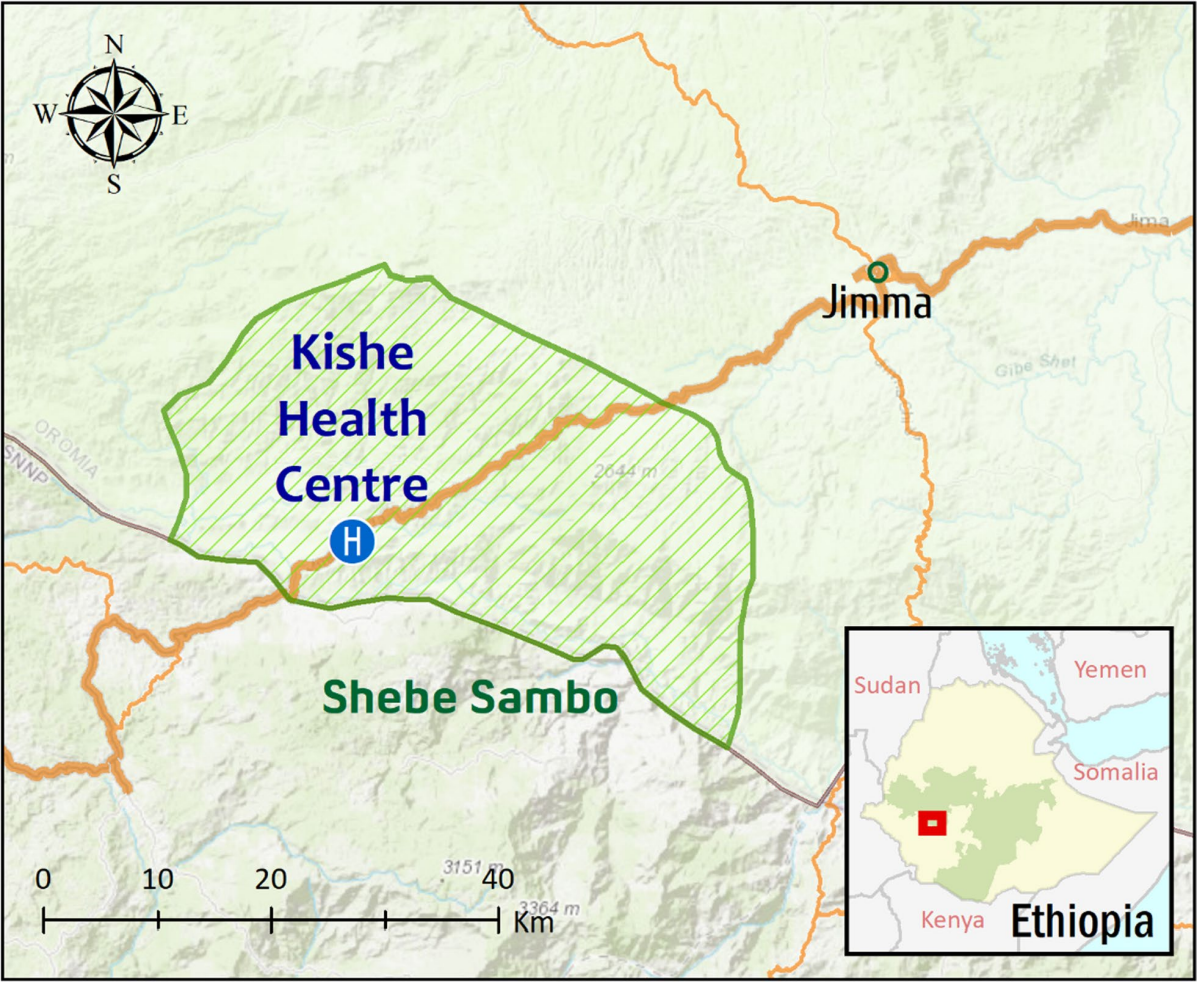
Map of the study area

The study was conducted in Kishe *kebele* located in Shebe Sambo district, Jimma Zone, Southwest Ethiopia. The district is located 52 km from Jimma Town, and 415 km south-west of Addis Ababa (Fig. 1). Approximate geographical coordinates of the district are 7°30′14″N, 36°30′44″E. Most of the inhabitants in Kishe settled before three decades, and there are also some indigenous people. Historical data shows that malaria is endemic in the area [8]. However, according to the information obtained from Kishe health centre and a recent study [13], passively detected malaria cases in the health centre have declined remarkably in recent years. Malaria transmission in the area is seasonal, similar to other unstable transmission areas in Ethiopia. Transmission peaks following the major rainy season of June to September, with minor transmission taking place in April and May. While maize is commonly grown in the area, small-scale rice irrigation is also practiced.

### Study design

A longitudinal entomological monitoring was carried out from June 2016 to May 2018. Moreover, a cross-sectional survey was conducted in August 2018 to gather data on human activities related to possible exposure to mosquito bite, and usage of ITNs by the inhabitants.

### Entomological survey

Adult mosquitoes were collected using human landing catches (HLCs), Centres for Disease Control and Prevention (CDC) light traps (LTs) and Pyrethrum Spray Catches (PSCs) monthly for 24 months. The HLC was conducted overnight from 6:00 pm to 6:00 am, with simultaneous indoor and outdoor collections. Two houses were selected for HLC operation for two nights of collection in each house every month, yielding a total of eight person-nights (four indoor and four outdoor) each month. In the study area, the houses were with roof constructed of either corrugated iron sheet or grass thatched. The walls were made from wood and mud plastered. Houses with thatched roof were selected for HLC operation. A team of four trained entomology technicians involved in the HLCs. The indoor and outdoor mosquito collectors exchanged every hour. The mosquitoes were collected using mouth aspirator and hand-held torch, as they landed on exposed legs. Mosquitoes captured in each hour were kept in pre-labelled individual paper cups covered with mesh. The anopheline mosquitoes were morphologically identified using keys [14] in the morning, and individually kept in labelled Eppendorf tubes over silica gel for further analysis.

Mosquitoes were also collected using CDC LTs. The CDC LTs were set overnight in another two houses for two nights in each house every month (four indoor and four outdoor trap-nights per month). One house from the middle and the other from periphery of the village were selected for installing the CDC LTs. Houses with thatched roof and corrugated iron sheet were included for the CDC LT collection. Moreover, indoor resting mosquitoes were collected by PSC in the morning from 6:00 am to 7: 30 am in a total of 20 smokeless houses each month. Food items and animals were removed from each house, and window(s) were closed before the operation. Aerosol insecticide (Baygon, SC Johnson & Son Inc, Racine, Wisconsin, USA) was prayed in the room, after which the house was closed for 15 min. Knocked-down mosquitoes were retrieved from the sheets, and morphologically identified. The gonotrophic status of each female *Anopheles* mosquito was scored as either unfed, freshly fed, half gravid or gravid. The anopheline mosquitoes were preserved as described above.

### Parity determination

Parity of *An. gambiae* collected using HLCs was determined at the field site in the morning following overnight collection by ovarian dissection following the method described by Detinova [15].

### Laboratory processing

#### Molecular identification of the Gambiae complex

A sub-sample of *An. gambiae* s.l. was identified to species using polymerase chain reaction (PCR) according to the method of Scott et al. [16], with some modifications. Wings and legs of the specimens were used for genomic DNA extraction using Qiagen DNA extraction kit. Diagnostic primers specific for *An. arabiensis* AR: (5′-AAGTGTCCTTCTCCATCCTA-3′), *An. gambiae* s.s. GA (5′-CTGGTTTGGTCGGCACGTTT-3′) and *An. amharicus* (*An. quadriannulatus* species B) QD-b: (5′-AGTGTCCAATGTCTGTGAAG-3′) were used for amplification, together with the universal primer UN: (5′-GTGTGCCCCTTCCTCGATGT-3′). The amplification reaction (PCR mix) contained 1 μL of genomic DNA, 0.5 μL of each primer and 10 μL of DreamTaq PCR Master Mix (2x) (Thermo Scientific) in a total volume of 20 μL. The following reaction conditions were used for amplification: denaturation at 94 °C for 30 s (35 cycles), annealing at 50 °C for 30 s, and elongation at 72 °C for 45 s. Visualization of the PCR products was performed on 2% agarose gel after staining with ethidium bromide.

### Sporozoite ELISA

The preserved anopheline mosquitoes were cut between thorax and abdomen, the head-thorax portion was processed for CSP-ELISA [17]. The mosquitoes were prepared as follows: pool of ten anopheline mosquitoes of the same species collected using same method were crushed using mortar and pestle. It was thoroughly homogenized using grinding buffer (GB), and tested immediately. After labelling the plate template, the U-bottomed ELISA plates were coated with pre-prepared capture monoclonal antibodies of Pf, Pv-210 and Pv-247, and incubated for 30 min. The contents were emptied and the plate banged up side down. This was followed by addition of blocking buffer and incubation for 1 h. After removing the contents, the test samples and controls were loaded into their respective wells and incubated for 2 h. The wells were then emptied and washed using PBS-Tween-20. Pre-prepared peroxidise-labelled conjugate solution was added to each well and incubated for 1 h at room temperature. After aspirating the contents and washing three times, ABTS substrate was added to each well and incubated for 30 min. Finally, the wells were read using ELISA reader adjusted at 405 nm.

### Blood meal analysis

Fed anopheline mosquitoes collected using PSC and CDC LTs were processed for human and bovine blood meal source(s) using direct ELISA [18]. Briefly, the engorged abdomen of each mosquito was individually ground in labelled Eppendorf tube. It was homogenized using phosphate-buffered saline (PBS). ELISA plates were coated with the homogenate, positive and negative controls, and incubated for 2 h at room temperature. The contents were then emptied and wells washed using PBS-Tween-20. Host-specific conjugates were added to the wells. Peroxidase-conjugated anti-human IgG and phosphatase-conjugated anti-bovine antibodies were added to the wells, followed by 1 h of incubation. After discarding the contents and washing the wells, 100 µl of ABTS substrate was added and incubated for 30 min. Intensity of the color produced was measured for human blood meal using ELISA reader at 414 nm. For detection of bovine blood meal, the same plates were washed, 100 µl pNPP added and incubated for 30 min. The development of color was measured as indicated above.

### Cross-sectional survey

Nocturnal human activities potentially exposing to mosquito bites, and utilization of ITNs by household members were assessed in a cross-sectional survey conducted in August 2018. The months June to September represent the major rainy season in the area, and malaria transmission usually peaks following this rainy season. Sample size for the cross-sectional survey was estimated using the general formula for single population proportion with the following assumptions: coverage of ITN to be 70.9% from a previous study in Jimma Zone [19], 95% confidence level and 5% margin of error. After adding 10% for anticipated non-response rate, the final sample size was calculated to be 349 households. A total of 1812 households were present in Kishe *kebele* during the study year. Systematic sampling technique was employed to select the households after obtaining list of the households for the *kebele* administration.

Pre-tested questionnaire developed for this study (Additional file 1) was used to collect the data. The questionnaire was first developed in English, translated to the local language (*Afan Oromo*) and back translated to English. The question items included house characteristics, demographic characteristics of household members, whether the household members stay outdoor after 6 pm and reasons for staying outdoor. Moreover, the number of ITNs owned by each household, usage of ITNs by each household member the preceding night and reasons for not using ITNs were assessed.

### Data analysis

Monthly entomological data were recorded in Microsoft Excel. Parous rate of *An. gambiae* s.l. was calculated as the ratio of parous anopheline to the total number of anopheline dissected. Access to ITNs was considered “sufficient” when a household has at least one ITN for every two members of the household [20]. Differences in mean indoor and outdoor anopheline density of HLC and CDC LT collections were compared using t-test. Seasonal difference in mean anopheline density was compared using one-way ANOVA from log-transformed data. Univariate and multivariable logistic regression were used to identify factors associated with ITN usage by the inhabitants. Variables with significant association and those with p-value < 0.2 during the univariate analyses were candidates for the multivariable model. Statistical significance was set at p < 0.05. The statistical analyses were performed using SPSS version 20 (Chicago, IL, USA).

## Results

### Species composition and abundance of anopheline mosquitoes

A total of 3659 anopheline mosquitoes belonging to four species were collected. *Anopheles coustani* complex was the predominant species (84.4%), followed by *An. gambiae* s.l (11.3%). Other anopheline species include *An. pharoensis* and *An. squamosus,* representing less than 5% of the total collections. Majority of the anopheline mosquitoes were collected using HLC (63.2%) followed by CDC LTs (36.5%) Table 1.

**Table 1:** Abundance and behavior of the anopheles mosquito species by method of collection in Kishe, Southwest Ethiopia, 2016–2018

Collection method	Collection location	Anopheline species				Total
		*An. coustani*	*An. gambiae* s.l	*An. pharoensis*	*An. squamosus*	
HLC	Indoor	904 (90.0)	81 (8.1)	17 (1.7)	2 (0.2)	1004 (27.4)
	Outdoor	1175 (89.8)	109 (8.3)	19 (1.5)	5 (0.4)	1308 (35.7)
CDC LT	Indoor	111 (56.1)	71 (35.9)	11 (5.6)	5 (2.5)	198 (5.4)
	Outdoor	897 (78.8)	144 (12.7)	81 (7.1)	16 (1.4)	1138 (31.1)
PSC		1 (9.1)	10 (90.9)	0 (0.0)	0 (0.0)	11 (0.3)
Total		**3088 (84.4)**	**415 (11.3)**	**128 (3.5)**	**28 (0.8)**	**3659 (100)**

Numbers in brackets represent percent

**Fig. 2:**
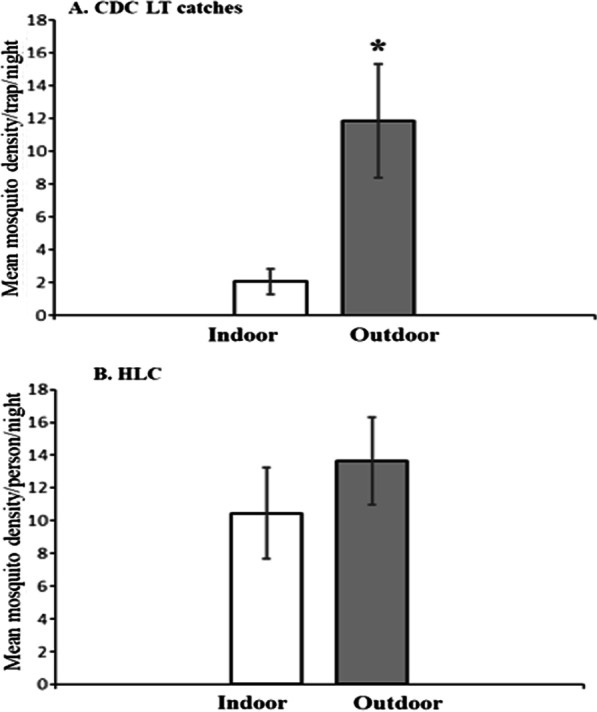
Mean anopheline mosquito density collected indoor and outdoor using CDC & HLC methods, Kishe, Southwest Ethiopia. Error bars indicate 95% confidence interval. *Significant at p < 0.05

Density of anopheline mosquitoes collected indoor and outdoor using HLC and CDC LTs is presented in Fig. 2. Overall, about two-thirds (67%) of the anopheline collected using CDC LTs and HLCs were captured outdoor. The mean mosquito density collected outdoor using CDC LTs was 11.8 mosquitoes/trap/night, which was significantly higher than those collected indoor (2.1 mosquitoes/trap/night) (p < 0.001). The mean anopheline density collected outdoor using HLC was 13.6 mosquitoes/person/night while mean mosquito density collected indoor was 10.5 mosquito/person/night.

**Fig. 3:**
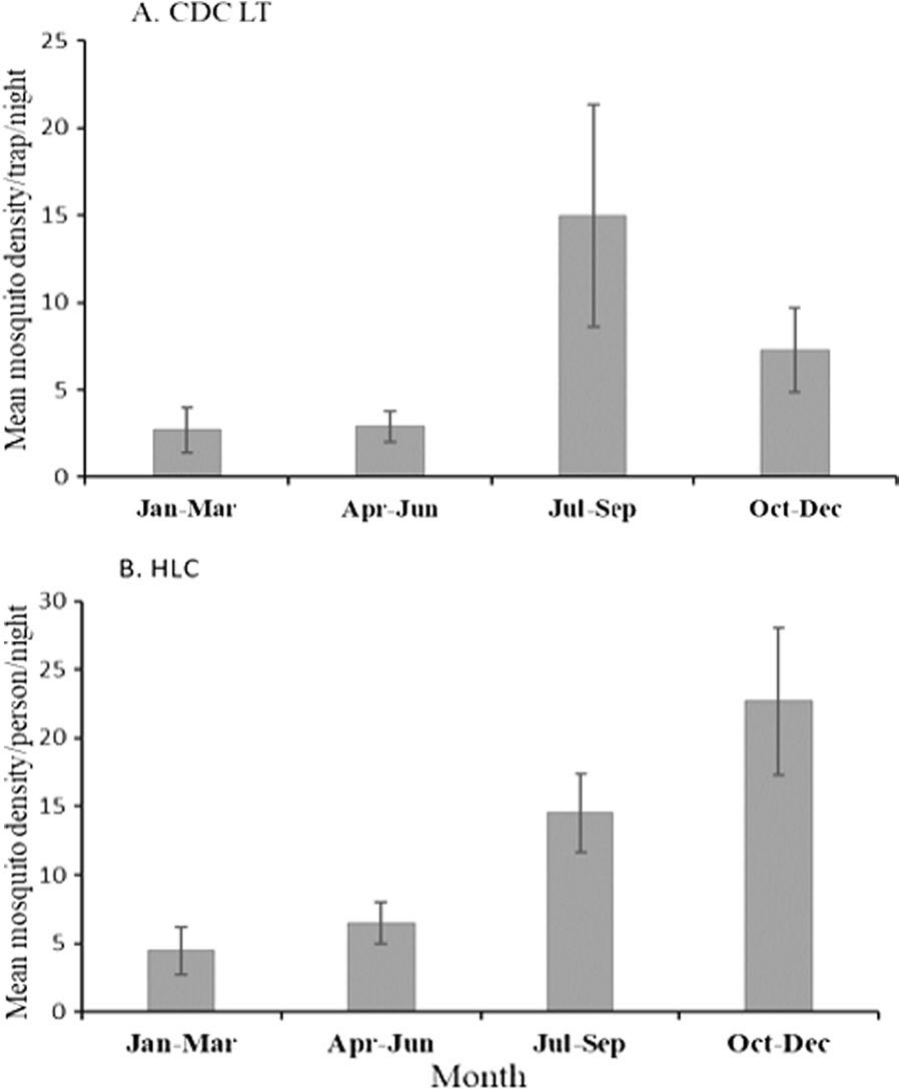
Mean anopheline mosquito density collected in different months using CDC LT and HLC methods in Kishe, Southwest Ethiopia. Error bars indicate 95% confidence interval

The mean anopheline mosquito density collected using CDC LTs and HLC in different months is presented in Fig. 3. Overall, there was significant seasonal difference in mean anopheline density collected using CDC LTs (F_(3,188)_ = 16.4 p < 0.001) and HLC (F_(3,188)_ = 39.4 p < 0.001). The mean density of anopheline mosquitoes collected in Jul-Sep using CDC LT was significantly higher (p < 0.05) than anopheline mosquito density collected in other months, and the mean anopheline mosquito density in Oct-Dec was significantly higher (p < 0.05) than those collected in the months of Jan-Mar. Similarly, pairwise comparison from HLC collections showed that, with the exception of Jul-Sep and Oct-Dec months, there was significant difference in mean anopheline mosquito density among seasons (p < 0.05).

### Nocturnal biting activities

**Fig. 4:**
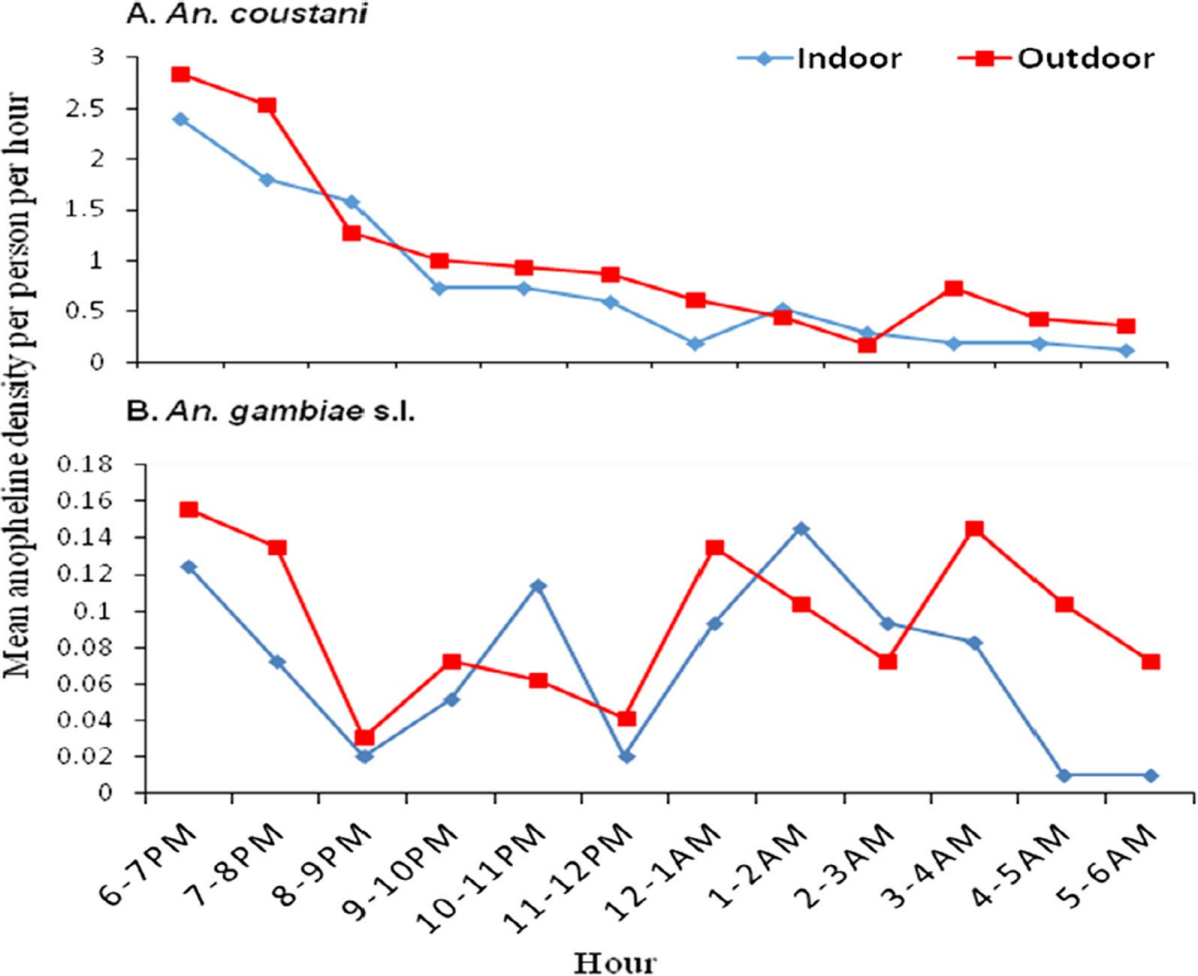
Mean hourly biting activity of anopheline mosquito species collected indoor and outdoor, Kishe, Southwest Ethiopia

Biting cycle of the anopheline mosquitoes is presented in Fig. 4. Majority of the anopheline mosquitoes (63%) aggressively bite in early hours of the night before most of the residents sleep (before 10 pm). The mean human biting rates for *An. coustani* from HLC was 10.8 bites/person/night, with slightly higher biting rate from outdoor HLC collection (12.2 bites/person/night) compared to indoor HLC (9.4 bites/person/night). The mean biting rate of *An. gambiae* s.l. was nearly 2 bites/person/night (0.84 bites/person/night and 1.13 bites/person/night indoor and outdoor, respectively).

### Species identification and parity of *An. gambiae* s.l.

A sub-sample of 100 *An. gambiae* s.l. specimens was randomly selected from different months and trapping methods, and tested for species identification using PCR. Ninety-three (93%) of the *An. gambiae* s.l. specimens were successfully amplified, all the amplified specimens were *An. arabiensis*. On the other hand, 68% (116/171) of the disected *An. gambiae* s.l. specimens were parous. Parous rate of *An. gambiae* s.l. before and after IRS operation was 75% and 17.5%, respectively. The proportion of parous *An. gambiae* s.l. significantly declined following IRS operation (p < 0.05).

### Sporozoite rate and host preference

Circumsprozoite protein of *P. falciparum* and *P.vivax* in all the anopheline mosquitoes collected was assayed using ELISA. None of the anopheline mosquitoes were positive for the tested *Plasmodium* CSPs. On the other hand, analyses of blood meal origins of the anopheline mosquitoes showed that *An. coustani* and *An. pharoensis* predominantly fed on cattle, with bovine blood index of 86.6% and 76.5%, respectively (Table 2). *An. gambiae* s.l. showed plasticity in its host preference. Half of the *An. gambiae* s.l. were anthropophagic, and most of the remaining half either fed on cattle or both cattle and human.

**Table 2:** Blood meal origin of the *Anopheles* mosquito species, Kishe, Southwest Ethiopia, 2016–2018

Species	# Tested	Blood meal origin			
		Human n (%)	Bovine n (%)	Mixed n (%)	Unknown n (%)
*An. coustani* complex	127	7 (5.5)	110 (86.6)	7 (5.5)	3 (2.4)
*An. gambiae* s.l	40	21 (52.5)	11 (27.5)	6 (15.0)	2 (5.0)
*An. pharoensis*	17	4 (23.5)	13 (76.5)	0 (0.0)	0 (0.0)
**Total**	**184**	**32 (17.4)**	**134 (72.8)**	**13 (7.1)**	**5 (2.7)**

Mixed: human and bovine blood

### Determinants of ITN usage

The cross-sectional survey included a total of 1351 individuals residing in 319 households. The majority of households (97.8%, n = 312) owned at least one ITN. However, only 62.4% (n = 199) had sufficient access to the ITNs (Additional file 2). Insecticide-treated net usage the preceding night before the survey was significantly higher among children under-five years of age (AOR = 7.9, 95% CI: 4.41–14.03) and household heads and spouses as compared to other family members (AOR = 4.8, 95% CI: 3.0–7.59). Moreover, individuals living in houses with sufficient access to ITNs (AOR = 1.8, 95% CI: 1.39–2.35) and those who do not use alternative mosquito repellents (AOR = 2.2, 95% CI: 1.58–2.99) had significantly higher odds of using ITNs (Table 3). The most commonly cited reasons for not using ITNs in the night preceding the date of survey (by those who did not use the ITNs) were assuming low risk of malaria (47.4%), scarcity or lack of ITNs (22%) and space not suitable to hang the ITNs (18.9%). On the other hand, most of the inhabitants (60.4%, n = 816) usually retire to bed after 9 pm and few (6.5%, n = 88) wake up before 6 am.

**Table 3:** Determinants of ITN usage by the inhabitants, Kishe, South west Ethiopia

Characteristics		Total n (%)	Individuals who used ITNs the preceding night n (%)	COR (95%CI)	AOR (95%CI)
Age (years)	< 5	160 (11.8)	146 (91.3)	4.3 (2.42–7.46)	7.9 (4.41–14.03)*
	≥ 5	1191 (88.2)	846 (71.0)	Ref	Ref
Sex	Male	669 (49.5)	478 (71.4)	Ref	
	Female	682 (50.5)	514 (75.4)	1.2 (0.96–1.56)	
Relationship to household head	Household head/spouse	558 (41.3)	477 (85.5)	4.0 (2.54–6.25)	4.8 (3.0–7.59)*
	Son/daughter	684 (50.6)	450 (65.8)	1.3 (0.86–1.97)	1.2 (0.76–1.80)
	Other relative	109(8.1)	65 (59.6)	Ref	Ref
Access to ITN	Sufficient	743 (55.0)	587 (79.0)	1.9 (1.48–2.41)	1.8 (1.39–2.35)*
	Not sufficient	608 (45.0)	405 (66.6)	Ref	Ref
Number of rooms	One	824 (61.0)	629 (76.3)	1.5 (1.14–1.86)	
	Two or more	527 (39.0)	363 (68.9)	Ref	
Family size	Less than four	266 (19.7)	218 (82.0)	1.8 (1.30–2.56)	
	Four or more	1085 (80.3)	774 (71.3)	Ref	
Malaria in the preceding 1 year	No	1320 (97.7)	969 (73.4)	Ref	
	Yes	31 (2.3)	23 (74.2)	1.0 (0.46–2.35)	
Use of alternative mosquito control**	No	1109 (82.1)	846 (76.3)	1.58–2.83	2.2 (1.58–2.99)*
	Yes	242 (17.9)	146 (60.3)	ref	Ref

*Significant at p < 0.05; **includes smoke and aerosol insecticide

## Discussion

Malaria control efforts have shown remarkable achievement in reducing morbidity and mortality in Ethiopia over the last two decades [21, 22]. This is likely due to effective vector control interventions, introduction of the potent artemisinin-based combination therapy and improved access to malaria diagnostics. The absence of sporozoite-infected anopheline in this study is, therefore, not unexpected, as one of the milestones of a successful vector control interventions is minimizing the transmission intensity [9, 23]. Similar CSP-negative findings were reported in recent years from some malaria endemic areas of Ethiopia [24–26]. The negative CSP result obtained in this study is likely attributed to the vector control interventions taking place in the area over the last decade. Indoor residual spraying in the study district has been supported by the President’s Malaria Initiative. Moreover, the community health extension program might have played important role in the use of core malaria preventive tools [27]. The odds of finding sporozoite positive sample is also related to the presence of parasite reservoirs in the human population infective to the vectors, which was low as shown in a recent report [13].

Overall, the mosquitoes collected outdoor were two-fold higher than those collected indoor. Similar findings of higher outdoor biting activity have also been reported elsewhere [7, 28]. The higher propensity of outdoor host seeking behaviour of the anopheline in the area poses a challenge for the anticipated malaria elimination. It calls for devising innovative vector surveillance and control methods targeting exophagic mosquitoes. Outdoor malaria transmission is a critical challenge in areas targeting malaria elimination. It might have been gradually triggered by long-term utilization of indoor-based vector control interventions [7]. Promising progress on improved outdoor surveillance tools [29] and interventions targeting outdoor biting *An. arabiensis* [30] were documented in recent years. Such outdoor-directed vector control tools need to complement the existing vector control tools to produce community-wide impact on malaria control and elimination. The higher anopheline mosquito density observed from July to September could be attributed to the main rains in the area during these months. While the months October to December are months when dry season starts in the area, the observed high density of anopheline during these months could be due to the small-scale irrigation activities taking place in the area.

Secondary and suspected vectors play crucial role in residual transmission of malaria. In this study, *An. coustani* complex was the most predominant mosquito species collected. The proportion of *An. gambiae* s.l. in this study (11.3%) was much lower than the proportion reported in a previous study conducted in a neighbouring district (69.7%) [26]. In this study, out of the sub-sample of *An. gambiae* s.l. specimens processed for molecular species identification, 93% were amplified. All of the amplified specimens were *An. arabiensis*. As the currently utilized vector control interventions are indoor-based, they mainly target the primary vector (*An. arabiensis*) of malaria in Ethiopia. This in turn allows relative proliferation of secondary and suspected vectors, which are implicated in sustaining low-level transmission of malaria [31]. The role of *An. coustani* complex in transmission of malaria in Ethiopia still remains unclear. It was implicated in outdoor transmission of malaria elsewhere [32]. Its zoophilic behaviour in the study area, which was also reported elsewhere in Ethiopia [33], could be one of the factors contributing to its CSP-negative finding. Most of the recent studies conducted in Ethiopia also reported either a negative or low CSP positivity in *An. coustani* complex [24, 34]. Secondary vectors of malaria are important in sustaining residual malaria transmission in areas achieving high coverage of the indoor-based vector control interventions [31].

Properly operated IRS deploying insecticide to which the local malaria vector is susceptible is essential in the prevention and control of malaria, and is the mainstay of malaria control in endemic countries. Taking parity of *An. gambiae* s.l as a proxy for vector longevity, significant decline in parous rate was observed post-IRS. Decline in parous rate is one of the key entomological indicators of effective IRS operation [35]. Longevity of the anopheline vector is a key component of its vectorial capacity, hence, affects transmission. The remarkable decline in parous rate of *An. gambiae* s.l following IRS operation in the area suggests the importance of this key vector control intervention. Indoor residual spraying primarily acts by either killing susceptible anopheline mosquitoes resting on sprayed structures, or repelling them before tarsal contact, ultimately reducing malaria transmission. Effectiveness of IRS is affected by the level of endemicity of malaria in the area, residual efficacy of the insecticide, susceptibility of the local mosquito vectors to the insecticide, IRS operational factors and concurrent utilization of other malaria control measures [36, 37].

Long-lasting insecticidal nets remain one of the frontline vector control tools widely used in malaria endemic countries. However, high coverage and proper utilisation of the nets is essential for sustained control of malaria. In this study, coverage of LLINs was remarkably high (97.8%) likely due to distribution of the nets three months prior to the survey. Although the coverage of LLINs was high, a sizable proportion of the inhabitants (26.6%) did not sleep under the nets the previous night before the survey. Nearly half (47.4%) of those who did not use the ITNs mentioned perceived low risk of malaria for not using the ITNs. Use of ITNs was significantly higher among children under-five years of age and household heads. Moreover, those individuals who responded to use smoke of certain plants as alternative mosquito repellent method appeared less likely to use ITNs. The use of such traditional smoke as an alternative mosquito repellent needs to be further investigated. With high coverage and proper utilization, ITNs confer community-wide protection [38], even in the face of outdoor transmission [39].

As ITNs are mainly used during the night, human night-time activities may affect their impact in the control of malaria. In this study, the most common reasons mentioned for staying outdoor after 6 pm include playing around peri-domestic areas by children, cooking or socializing by adult females and socializing or feeding animals by adult males. Some of these activities were also reported as reasons for staying outdoor in areas of high ITN coverage elsewhere [40]. The overlap in peak biting activity of the anopheline mosquito vectors early evening during which most of the inhabitants are not protected by ITNs likely puts the occupants at greater risk of infection. As majority of the households had kitchens separate from the house where the family members sleep, cooking earlier in the evening may also increase the likelihood of human-vector contact.

The following limitations of the study should be noted. Mosquitoes were collected from only two houses using HLC and another two houses using CDC LTs, which have limited the number of anopheline collected and processed to estimate the entomological indices. Moreover, due to resource constraints, small proportion of the *An. gambiae* s.l. specimens were processed for molecular species identification. Furthermore, human behavioural activities were assessed using limited quantitative data. Qualitative methods which help to better understand night-time human activities and sleeping patterns were not used in this study.

## Conclusion

Overall, the anopheline mosquito species in the area tend to bite predominantly outdoor and early in the evening. There was significant seasonal variation in the density of anopheline mosquitoes in the area. Parous rate of *An. gambiae* s.l. significantly declined following IRS operation, proving its vital role as a vector control intervention. *An. coustani* and *An. pharoensis* were predominantly zoophilic, and *An. gambiae* s.l. appeared to be opportunistic in its feeding behaviour. On the other hand, household ITN coverage was remarkably high with modest usage by the inhabitants. Strengthening the available vector control interventions and community sensitization on sustained use of ITNs is essential. Moreover, developing better surveillance and control methods targeting exophagic anopheline mosquito vector species is required.

## Supplementary Information

**Additional file 1.** Questionnaire developed to assess house characteristics of study participants. Questionnaire developed to assess characteristics of the household members.

**Additional file 2.** Household characteristics of the inhabitants in Kishe, Southwest Ethiopia.

## Data Availability

The data supporting the results reported in this article are included within the article and its additional files.

## Abbreviations

ANOVA: Analysis of variance
CDC LT: Centers for Disease Control and Prevention Light Trap
CI: Confidence interval
CSP: Circumsporozoite protein
ELISA: Enzyme-linked immunosorbent assay
HLC: Human landing catch
IRS: Indoor residual spraying
LLIN: Long-lasting insecticide-treated net
OR: Odds ratio
PBS: Phosphate-buffered saline
PCR: Polymerase chain reaction
PSC: Pyrethrum spray catch
