# Supplementary material for: Malaria vector dynamics and utilization of insecticide-treated nets in low-transmission setting in Southwest Ethiopia: implications for residual transmission
Source: BMC Infect Dis. 2021 Aug 28;21:882. doi: 10.1186/s12879-021-06592-9 (PMC8403392; doi:10.1186/s12879-021-06592-9)
Supplement: Supplementary file 1 — Additional file 1. Questionnaire developed to assess house characteristics of study participants. Questionnaire developed to assess characteristics of the household members. [file 12879_2021_6592_MOESM1_ESM.docx]

**Additional file 1**

1. **Questionnaire developed to assess house characteristics of study participants**

**House code_________**

| **Q.no** | **Questions** | **Response** | **Code** |
| --- | --- | --- | --- |
| **A1** | Roof structure | 1. Corrugated iron sheet 2. Thatched |  |
| **A2** | Ceiling | 1. Present 2. Absent |  |
| **A3** | Presence of window | 1. Yes 2. No |  |
| **A4** | Window screening | 1. Yes 2. No |  |
| **A5** | Eave gap | 1. Present 2. Absent |  |
| **A6** | Visible hole on the house wall | 1. Yes 2. No |  |
| **A7** | Number of sleeping rooms within the house | 1. One 2. Two 3. Three or more |  |
| **A8** | Location of kitchen | 1. Within the living room 2. Separate from the main house 3. Separate, but also within the living room |  |
| **A9** | Family livelihood mainly depends on: | 1. Farming 2. Trade 3. Others (specify)_____ |  |
| **A10** | Type of habitation | 1. Human only 2. Mixed (human and animals in the same house) |  |
| **A11** | Do you have cattle? | A. Yes   1. B. No |  |
| **A12** | If yes, do you keep them in cowshed? | A. Yes  B. No |  |
| **A13** | Do you have ITN(s)? | 1. Yes 2. No |  |
| **A14** | If yes, how many ITN(s) do have? |  |  |
| **A15** | Mention if you use any other mosquito prevention method in your house |  |  |

| **S. no** | **Questions** | **Response** | **Code** |
| --- | --- | --- | --- |
| **B1** | How old are you? |  |  |
| **B2** | Sex | 1. Male 2. Female |  |
| **B3** | Relationship to the household head | 1. Spouse 2. Son/daughter 3. Other relative |  |
| **B4** | What is your current occupation? | 1. Farmer 2. Housewife 3. Student   D. Others (specify)______ |  |
| **B5** | What was the highest level of schooling you attended? |  |  |
| **B6** | Did you have malaria in the preceding 12 months? | 1. Yes 2. No |  |
| **B7** | Did you sleep under ITN last night? | 1. Yes 2. No |  |
| **B8** | If no, mention your reason(s) |  |  |
| **B9** | At what time did you sleep last night? |  |  |
| **B10** | What is your usual sleeping time? |  |  |
| **B11** | At what time did you wake up this morning? |  |  |
| **B12** | At what time do you usually wake up in the morning? |  |  |
| **B13** | Do you usually stay outdoor earlier in the evening (after 6 pm)? | 1. Yes 2. No |  |
| **B14** | If yes, mention your reason(s)? |  |  |

**B. Questionnaire developed to assess characteristics of the household members**
