# Supplementary material for: Malaria vector dynamics and utilization of insecticide-treated nets in low-transmission setting in Southwest Ethiopia: implications for residual transmission
Source: BMC Infect Dis. 2021 Aug 28;21:882. doi: 10.1186/s12879-021-06592-9 (PMC8403392; doi:10.1186/s12879-021-06592-9)
Supplement: Supplementary file 2 — Additional file 2. Household characteristics of the inhabitants in Kishe, Southwest Ethiopia. [file 12879_2021_6592_MOESM2_ESM.docx]

**Additional file 2. Houshold characteristics of the inhabitants in Kishe, Southwest Ethiopia**

| House characteristic (n=319) | Frequency | Percent |
| --- | --- | --- |
| Roof structure |  |  |
| Iron sheet | 190 | 59.6 |
| Thatched | 129 | 40.4 |
| Number of rooms |  |  |
| One | 211 | 66.1 |
| Two or more | 108 | 33.9 |
| Visible hole on house wall |  |  |
| No | 273 | 85.6 |
| Yes | 46 | 14.4 |
| Eave gap |  |  |
| Present | 61 | 19.1 |
| Absent | 258 | 80.9 |
| Ceiling |  |  |
| Absent | 210 | 65.8 |
| Present | 109 | 34.2 |
| Presence of window |  |  |
| No | 126 | 39.5 |
| Yes | 193 | 60.5 |
| Window screening |  |  |
| No | 311 | 97.5 |
| Yes | 8 | 2.5 |
| Location of kitchen |  |  |
| Separate***** | 305 | 95.6 |
| Within the living house only | 14 | 4.4 |
| Habitation type |  |  |
| Mixed | 55 | 17.2 |
| Human only | 264 | 82.8 |
| Own at least one ITN |  |  |
| Yes | 312 | 97.8 |
| No | 7 | 2.2 |
| Access to ITN |  |  |
| Not sufficient/not available | 120 | 37.6 |
| Sufficient | 199 | 62.4 |
| Use of other mosquito repellents |  |  |
| No | 266 | 83.4 |
| Yes** | 53 | 16.6 |
| Family livelihood mainly depends on |  |  |
| Farming | 291 | 91.2 |
| Others*** | 28 | 8.8 |
| Family size |  |  |
| ≤4 | 116 | 36.4 |
| >4 | 203 | 63.6 |

*include houses with a kitchen structure separate from the main house ** Smoke from certain plants and aerosol insecticides ***include trade, salary/employment, driver and daily laborer
